# Heparin‐induced hyperkalemia, can LMWH cause hyperkalemia? A systematic review

**DOI:** 10.1002/jha2.801

**Published:** 2023-10-03

**Authors:** Gedion Yilma Amdetsion, Aron Gudeta, Guy Lumley, Harkiran Sagoo, Ehab Aliledhin

**Affiliations:** ^1^ Internal Medicine Division Addis Ababa University Addis Ababa Ethiopia; ^2^ Trauma and Orthopaeics Division Barts Health NHS Trust London UK

**Keywords:** enoxaparin, HEPARIN, heparin induced hyperkalmeia, hyperkalemia, hypoaldosteronism, LMWH

## Abstract

Hyperkalemia, an elevated blood potassium concentration exceeding 5.0 mEq/L, is associated with adverse outcomes and is frequently observed in hospitalized patients. Drug‐induced hyperkalemia accounts for a significant proportion of cases, with heparin, commonly used for venous thrombosis prevention, suspected to contribute, though less recognized than other heparin‐related side effects. Both unfractionated heparin (UFH) and low molecular weight heparin (LMWH) have been implicated in inducing hyperkalemia, primarily through the suppression of aldosterone levels and modulation of angiotensin II receptors. This systematic review examines the relationship between heparin, particularly LMWH, and hyperkalemia. Thirteen studies involving 1407 patients were analyzed. Findings indicated a lack of highquality evidence, with no significant increase in potassium levels associated with LMWH use. LMWH did not exhibit a dose‐response relationship with hyperkalemia incidence. Additionally, mechanisms underlying the hypothetical LMWHinduced hyperkalemia remained inconclusive. While this suggests that LMWH is unlikely to be a primary cause of hyperkalemia, caution is warranted, especially in patients with elevated baseline potassium levels.

## Abstract 2

Can LMWH cause Hyperkalemia: A systematic review: LMWH is unlikely to cause hyperkalemia, but caution is advised, especially in patients with high baseline potassium.

## 1 INTRODUCTION

Hyperkalemia refers to an elevated concentration of potassium in the blood, specifically when it exceeds 5.0 mEq/L. This condition has been linked to various negative outcomes, including increased mortality rates and higher healthcare expenses [1, 2]. Such association has been observed across different clinical situations, particularly among hospitalized individuals. The primary risk factors for hyperkalemia include chronic kidney disease, metabolic acidosis, and certain medications. In fact, drug‐induced hyperkalemia is responsible for approximately 75% of patients with hyperkalemia admitted to hospitals [3].

While Heparin is commonly used for preventing venous thrombosis in various inpatient hospital departments, its association with hyperkalemia is not as widely recognized by doctors as its connection to other side effects like heparin‐induced thrombocytopenia and skin necrosis. Both unfractionated heparin (UFH) and low molecular weight heparin (LMWH) have long been suspected to induce hyperkalemia in a subset of patients undergoing anticoagulation treatment. This effect is primarily caused by the suppression of aldosterone levels, both directly and indirectly by influencing the number and affinity of angiotensin II receptors [4, 5]. Heparin can lead to hyperkalemia on its own, but the risk is further increased when used in conjunction with medications that can predispose to hyperkalemia like angiotensin converting enzyme (ACE) inhibitors, nonsteroidal anti‐inflammatory drugs (NSAIDs), and trimethoprim [6]. This mechanism of potassium elevation is usually compensated for in healthy individuals but is more pronounced in elderly individuals, diabetics, and those with renal failure, especially when taking the aforementioned medications. The risk of hyperkalemia is also influenced by higher doses, prolonged usage, and the choice of unfractionated heparin therapy [7].

Since the 1960s, it has been established that heparin has an impact on adrenal gland metabolism. Animal experiments have shown that this interference depends on the dosage and duration of heparin administration [8]. The peak of this interference occurs around 4–5 days after heparin application and remains effective for up to 7 days, as confirmed in human studies [9]. Heparin primarily affects the adrenal zona glomerulosa, particularly inhibiting steroidogenesis at the 18‐hydroxylase stage. This suppression is observable both in primary and secondary hyperaldosteronism [9, 10]. Prolonged use of heparin over several years can lead to atrophy of the zona glomerulosa [11]. As a consequence, hypoaldosteronism may develop, potentially causing hyperkalemia, especially in patients with existing predispositions for impaired adrenal aldosterone production, such as those with diabetes mellitus or renal insufficiency.

This study is the first systematic review looking into the relationship between LMWH and hyperkalemia. Its primary aim is to establish the relationship between hyperkalemia and heparin use. It was done to attempt to identify the incidence, confirm whether the aforementioned factors do indeed increase the likelihood of heparin‐induced hyperkalemia, and, if possible, establish medical comorbidities that increase the likelihood for heparin‐induced hyperkalemia.

## 2 METHOD

We conducted an online systematic literature search in accordance with the Preferred Reporting Items for Systematic Reviews and Meta‐Analyses (PRISMA) guidelines. It was prospectively registered on the International Prospective Register of Systematic Reviews (PROSPERO: CRD42023445658)

We systematically searched for studies reporting the presence and absence of hyperkalemia in patients taking heparin in MEDLINE, EMBASE, and Cochrane Library databases from inception to 04/08/2023. We conducted the search using this terms ((“Heparin, Low‐Molecular‐Weight”[Mesh]) OR (“Heparin”[Mesh])) and outcome ((((“Hyperkalemia”[Mesh]) OR (“Potassium”[Mesh])))). No language restriction was applied. We included all studies conducted in adults above 18 years of age who are taking heparin (UFH/LMWH) and evaluate the rate of hyperkalemia incidence. We included studies that case‐control, retrospective and prospective cohorts, and randomized controlled trials (RCTs). Two authors independently reviewed and screened the abstracts based on the specified inclusion/exclusion criteria. The full text of the selected articles was also independently reviewed and screened by two authors, with any discrepancies once again resolved by the senior author. After performing Risk of bias assessment using ROBINS‐I tool for nonrandomized studies (controlled/cohort/case‐control, etc.): ROBINS‐I tool. And for Randomized studies we used RoB 2 tool. Then Data were extracted in a standardized format by two authors.

## 3 RESULT

**FIGURE 1:**
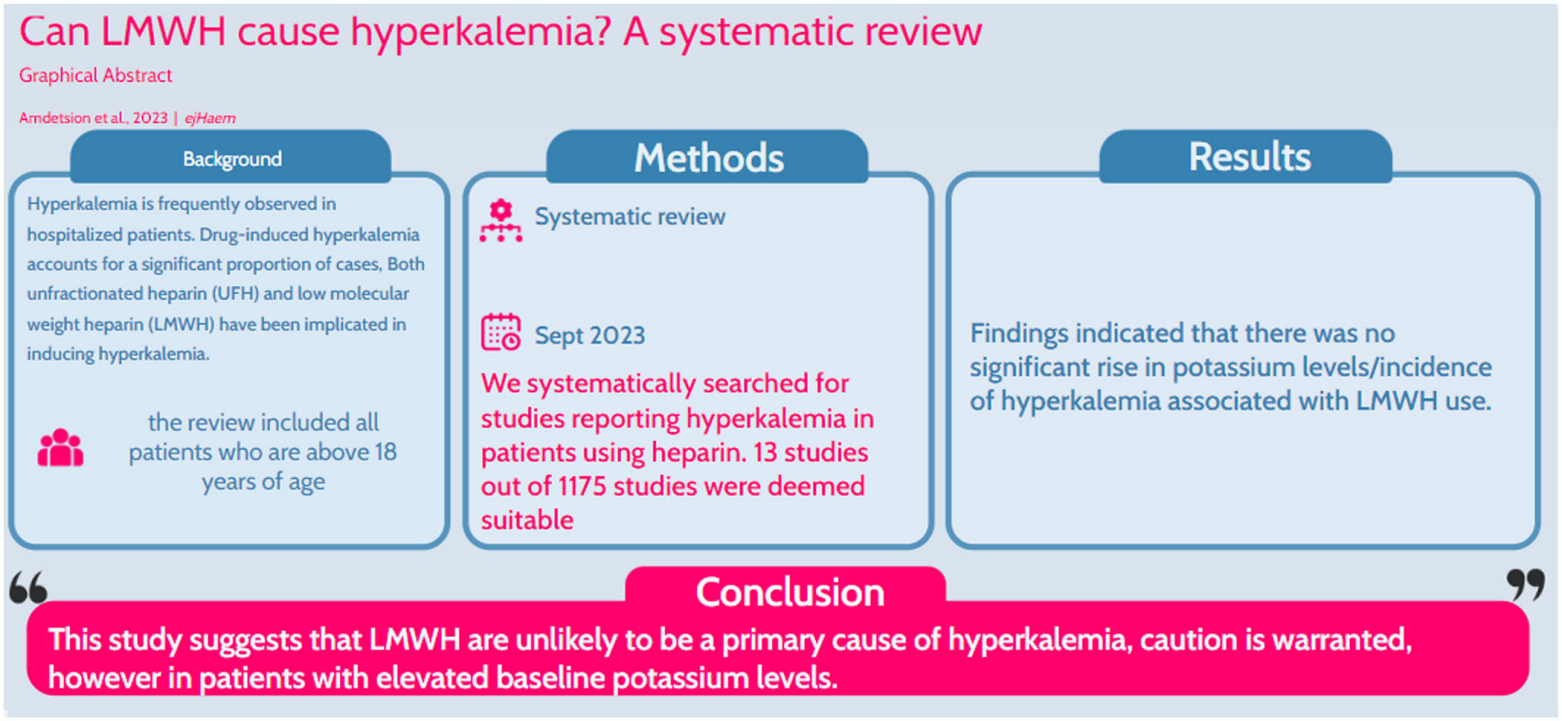
Risk of bias assessment.

Following the utilization of the aforementioned search items, a comprehensive tally of 1175 studies was identified. Subsequently, through the meticulous application of our inclusion and exclusion criteria, a total of 13 relevant studies were deemed suitable for our review. Upon thorough examination of the reference lists and author contacts associated with these pertinent articles, no additional studies were uncovered. Independent analysis of the complete texts of all the identified articles ultimately yielded no further studies that could be incorporated into the final review. Amongst the selected studies, seven constituted cohort studies, while two was a posthoc analysis of a clinical trial, and the remaining ones were RCT. We then proceeded to do Risk of bias assessment using Murad et al. 2018 for case series and ROBINS‐I tool for nonrandomized studies (controlled/cohort/case‐control etc.) (Figure 1).

The analysis involved a narrative synthesis of the study findings, organized according to the type of intervention, specific characteristics of the target population and the outcome assessed. Unfortunately we were unable to perform quantitative analysis due to the heterogeneity of the included studies. However, we did try to perform combined mean whenever feasible to better elaborate the results (Table 1).

**TABLE 1:** Summary of the included researches.

Study	Country	Research type	Number of patients	Age (S±)	Baseline potassium	Potassium level after treatment
Ezzatzadegal et al. [12]	Iran	RCT	58:			
			UFH: 29	52.8 ± 15	4.9 ± 0.7 mEq/L	4.8 ± 0.5
			Enoxaparin: 29	55.6 ± 17	4.9 ± 0.8 mEq/L	4.4 ± 0.5
Merzer et al. [13]	Germany	Post hoc analysis	117			
			Placebo: 58	57.5 ± 11.2	4.3 ± 0.4	4.2 ± 0.4
			Certoparin: 59	60.0 ± 10.0	4.2 ± 0.4	4.2 ± 0.4
Abdel‐Raheem et al. [14]	US	Cohort	28	70	4.25 ± 0.40	4.35 (± 0.41)
Gheno et al. [15]	Italy	Cohort	416	73 ± 12	4.2 ± 0.5 mmol/l	4.5 ± 0.5 mmol/l
			Nadoprine: 285			
			Enoxaparin: 131			
Bengalorkar et al. [16]	India	RCT	60			
			UFH: 30	56.40 ± 16.08	4.09 ± 0.49	4.17+_0.65
			Enoxaparin: 30	57.55 ± 12.67	4.29 ± 0.46	4.32+_ 0.75
Torres et al. [17]	Spain	Prospective cohort study	246	80.5 ± 12.1	4.1 ± 0.5	4.3 ± 0.5
			Bemparin: 108			
			Bemiparin: 138			
Rocha et al. [18]	Spain	Post hoc analysis of clinical trial	215			
			UFH: 104	57.69 ± 15.15	4.35 ± 0.40	4.54 ± 0.45
			Bemparin: 111	56.81 ± 16.33	4.32 ± 0.43	4.46 ± 0.41
Koren‐Michowitz et al. [19]	Israel	Cohort study	85	62 ± 11.8	4.26 ± 0.04	4.43 ± 0.04
Potti et al. [20]	US	Cohort study	78	67	4.01 ± 0.30	4.37 ± 0.47
Hottelart et al. [21]	France	RCT: crossover	12	57.2		
			UH (6160 IU ± 1350 IU): 2 weeks			5.66 ± 0.83
			LMWH (15 antiXa activity [aXa] U/kg + 5 aXa U/kg/h): 2 week			5.16 ± 0.68
K. S Wong et al. [22]	US	RCT	Total: 270	Not provided		
			Placebo : 88		3.86 (0·54)	4.23 (0.52)
			Low dose nadoprine: 91		3.84 (0.52)	3.93 (0.54)
			High dose: 91		3.80 (0.49)	4.10 (0.55)
Canova et al. [23]	Switzerland	Cohort	Total: Nadoprine 81	Not provided	4.06 ± 0.38	4.25 ± 0.40
			No renal disease		4.01 ± 0.35	4.17 ± 0.37
			Renal		4.26 ± 0.57	4.59 ± 0.62
			diabetes		4.1 ± 0.25	4.16 ± 0.27
			ACE inhibitor		4.18 ± 0.4	4.42 ± 0.36
			NSAID		3.98 ± 0.46	4.29 ± 0.39
Siebels et al. [24]	Germany	RCT	29			
			UFH 2 × 5000	25		
			UFH 3 × 5000	59		
			UFH 3 × 7500	62	3.7	4.5
			LMWH 1 × 2500	25		
			LMWH 1 × 5000	53		

Abbreviation: ACE, angiotensin converting enzyme.

### 3.1 Demographics

There were a total of 1409 patients in the study. Among the research reports, Rocha et al. [18], Canova et al. [23], and Wong et al. [22] did not provide sex ratio data. However, from the reports that did mention the sex ratio, 54% were male and 46% were female. The average age range was 67.3306 ± 2.9417.

### 3.2 Potassium

In relation to baseline potassium levels for patients using LMWH, focusing on studies that documented this data and excluding those undergoing hemodialysis, we found a sample of 1351 patients. Their initial potassium level averaged at 4.1825 ± 0.1974. Meanwhile after they were on LMWH, the potassium level slightly rose to 4.3438 ± 0.02. Although this increase was not statistically significant, it did indicate a slight elevation in potassium levels.

During the stratified analysis, a total of 357 patients on Bamiparine were identified across two studies. In both of these studies, there was no statistically significant increase in potassium levels when their means were combined. Initially, the baseline potassium level was 4.17 ± 0.37, which then increased to 4.452 ± 0.378 For Nadoprine, a total of 263 patients were included from two studies (Wong et al. and Canova et al. [22, 23]). The baseline potassium level was 3.9 ± 0.273, which increased to 4.084 ± 0.293 during LMWH treatment. In the case of ENOXAPARIN, 278 patients from four studies (Potti et al., Koren‐Michowitz et al., Abdel‐Raheem et al. and Bengalorkar et al. [14, 16]) were combined. The initial potassium level was 4.1925 ± 0.1425, which elevated to 4.3855 ± 0.2 during the treatment phase.

Conversely, for Unfractionated Heparin (UFH), two studies (Rocha et al., and Bengalorkar et al. [16, 18]) involving 134 patients were included. The baseline potassium level was 4.29 ± 0.33. After the initiation of treatment, it increased to 4.46 ± 0.37.

In terms of hyperkalemia incidence, Bengalorkar et al. [16] found four cases in their LMWH group, where there was no significant association with comorbidities or baseline potassium levels. In the UFH group, there were two cases linked to baseline potassium levels. Torres et al. identified 14 hyperkalemia cases, with 10 confirmed, only three patients had K levels above 5.5, one with worsened renal function. ACE inhibitor use and baseline potassium were linked in their analysis. Melzer et al. [13], reported 10 cases of hyperkalemia, however it was probably linked to the other drugs as the patients were taking ACE inhibitors, and NSAIDS Rocha et al. [18] found no hyperkalemia with bemparin but 4 cases with UFH. Koren‐Michowitz et al. [19] noted eight mild hyperkalemia cases with ENOXAPARIN, potentially related to its nephrologic effects. Potti et al's [20] ENOXAPARIN study had zero hyperkalemia incidence and no significant trans tubular K gradient change either, contrary to Koren‐Michowitz et al. [19], although patients only received prophylactic doses.

Wong et al. [22] observed no significant potassium change with Nadoprine, and Canova et al. reported hyperkalemia linked to baseline potassium above 5 mmol/L.

Siebels et al. [24] studied aldosterone, renin, and electrolytes. LMWH (Fragmin) at both prophylactic and treatment doses did not suppress aldosterone, while UFH therapeutic doses did.

### 3.3 TTKG

While when it comes to transtubular potassium Gradient, we have identified 116 patients taking enoxaparin across two researches(Abdel‐Raheem et al., potti et al. [14, 20]), the baseline TTKG before starting enoxaparin was 5.5568 ± 1.807, while after treatment 5.9185 ± 2.34.

### 3.4 Comorbidity

In the LMWH group, various comorbidities were observed. Renal conditions were present in 23 patients, while 70 were undergoing haemodialysis. Additionally, 170 patients had diabetes. Conversely, in the UFH group, 25 patients had diabetes.

### 3.5 Aldosterone

Regarding aldosterone levels, when considering the baseline values from the two papers (Koren‐Michowitz et al. and Siebels et al. [19, 24]) collectively, they averaged around 13 ± 3.66. After treatment, this value decreased to 10.36 ± 6.6. However, when specifically analyzing ENOXAPARIN, its use led to an increase in aldosterone levels from 4.0 ± 0.48 to 6.9 ± 1.6.

Meanwhile for the UFH group in Siebels, they demonstrated aldosterone suppression in the combined LMWH, with the initial Aldosterone being 16.26 ± 3.983. However, after 4 days of treatment it became 10.2773 ± 2.7.

## 4 DISCUSSION

This systematic review was undertaken to evaluate whether heparin, specifically LMWH, contributes to hyperkalemia, along with exploring the potential mechanisms behind such a connection. The review encompassed data from thirteen studies involving 1407 participants. These studies exhibited considerable diversity in terms of patient comorbidities, the type of heparin administered, proposed mechanisms, and concluding statements.

The systematic review revealed four primary findings based on the analysis of the trials. Firstly, it was evident that there was a notable scarcity of high‐quality evidence within the available literature concerning the subject in question. Secondly, there was no statistically significant evidence to support the idea that enoxaparin, a type of LMWH, leads to a reduction in renal potassium secretion. Thirdly, while the data indicated that unfractionated heparin (UFH) might cause aldosterone suppression, the evidence for LMWH, particularly enoxaparin, was less robust. Lastly, LMWH was found to be associated with a significantly lower risk of causing hyperkalemia when compared to UFH. Unfortunately, the available evidence did not permit the aggregation of results for a more precise meta‐analysis.

The primary question our review aimed to address was whether LMWH leads to hyperkalemia. Among the 1351 patients included in the reviewed studies who were administered LMWH, their initial potassium levels or the combined mean potassium levels of the studies showed an increase from an average of 4.1825 ± 0.1974 to 4.3438 ± 0.12. The confidence interval between the initial and final potassium levels did not show a statistically significant difference, suggesting that the observed variation in the average potassium levels could be attributed to random chance. It is worth noting that in all the studies we included, there was a notable overlap in the confidence intervals for potassium levels before and after the initiation of LMWH treatment.

When we conducted a subgroup analysis, examining the combined mean potassium levels for different types of LMWH, there was no clear evidence of a significant increase in potassium levels, except for bemparin, which showed a noticeable rise in potassium levels.

Regarding the incidence of hyperkalemia, Bengalorkar et al. [16]. identified four cases in their LMWH group, even though they did not find a statistically significant increase in potassium levels. Interestingly, they could not pinpoint any definitive risk factors for this rise, but it was observed that hyperkalemia had a strong connection to baseline hyperkalemia. However, it is essential to consider that all these patients were admitted for acute coronary events, which often warrant the initiation of ACE inhibitors and betablockers—medications known to elevate potassium levels. Therefore, the results should be interpreted with caution.

Torres et al. [17] detected 14 cases of hyperkalemia, with 10 of them confirmed. Notably, only three patients had potassium levels exceeding 5.5. Furthermore, both multivariate and univariate analyses indicated that the most significant factor associated with hyperkalemia was the baseline potassium level.

Melzer et al. [13] reported 12 cases of hyperkalemia. However, this was likely attributed to other medications, as the patients were also taking ACE inhibitors and NSAIDs. Interestingly, there was a similar incidence of hyperkalemia in patients taking a placebo.

It is worth mentioning that the incidence of hyperkalemia does not appear to be correlated with the dosage of LMWH administered. Thus we took our study a bit deeper, to find out if LMWH indeed caused a hypoaldosteronism,

When we combined the baseline aldosterone values from both Koren‐Michowitz et al. and Siebels et al. [19, 24], we did not observe any statistically significant reduction. In fact, in the enoxaparin group, there was a notable increase in aldosterone levels. This led Koren‐Michowitz to suggest that LMWH might induce hyperkalemia, or at the very least, enoxaparin might do so due to its potential direct effect on the kidneys. As a result, we examined the transtubular potassium gradient (TTKG) of patients receiving LMWH, which showed a non‐significant increase in TTKG. However, it is essential to note that these TTKG levels did not correlate with either renal tubular acidosis type 4 or renal potassium mishandling.

It is also worth mentioning that although TTKG levels are typically below 7 in cases of hyperkalemia induced by hypoaldosteronism, among the patients for whom TTKG was calculated, none experienced hyperkalemia.

Taking all factors into account, the evidence suggests that LMWH does not exhibit a dose‐response relationship with the incidence of hyperkalemia. Moreover, the proposed mechanisms for LMWH‐induced hyperkalemia remain inconclusive. Therefore, it can be reasonably concluded that LMWH is unlikely to be a primary cause of hyperkalemia.

However, it is important to note that the studies included in this review were not uniform enough to perform a comprehensive meta‐analysis. Additionally, it is prudent to remain cautious, as hyperkalemia could still occur in specific cases, especially in patients with elevated baseline potassium levels.

## AUTHOR CONTRIBUTIONS

Amdetsion conceived of the presented idea. Amdetsion, Sagoo, and Gudeta developed the protocol. Lumley reviewed the protocol and edited it. Amdetsion, Sagoo, Aliledin, and Gudeta were involved in searching, selecting, and extracting data from the studies. The result was computed by Amdetison. The findings of this work were then discussed among all the authors. Then the manuscript was written by Amdetsion. It was then reviewed by Lumley and then the others.

## CONFLICT OF INTEREST STATEMENT

The authors have no conflict of interest to declare. All co‐authors have seen and agree with the contents of the manuscript, and there is no financial interest to report.

## FUNDING INFORMATION

The authors received no specific funding for this work and it was fully self‐funded.

## ETHICS STATEMENT

This systematic review used publicly accessible researches as evidence, with no interaction with patients or animals and thus an ethical approval was not required.

## CLINICAL TRIAL REGISTRATION

The authors have confirmed clinical trial registration is not needed for this submission.

## PATIENT CONSENT STATEMENT

The authors have confirmed patient consent statement is not needed for this submission.

## Data Availability

The authors confirm that the data supporting the findings of this study are available within the article and its listed references.
